# Supplementation with Chinese herbal preparations protect the gut-liver axis of Hu sheep, promotes gut-liver circulation, regulates intestinal flora and immunity

**DOI:** 10.3389/fimmu.2024.1454334

**Published:** 2024-11-13

**Authors:** Zilong Liu, Huihui Wang, Keyan Ma, Qiao Li, Yi Wu, Xingcai Qi, Juanjuan Song, Chunhui Wang, Youji Ma, Taotao Li

**Affiliations:** ^1^ College of Animal Science and Technology, Gansu Agricultural University, Lanzhou, China; ^2^ Gansu Key Laboratory of Animal Generational Physiology and Reproductive Regulation, Gansu Agricultural University, Lanzhou, China

**Keywords:** metabolism, Chinese herbal preparations, gut-liver axis, ileal microorganisms, immunity

## Abstract

The gut-liver axis in ruminants can explain nutrient regulation, the gut-liver cycle, and immune function in ruminant biology through the gut microbe-gut metabolite-liver metabolite relationship. to investigate the effects of herbal feed additives on the gut-liver axis of Hu sheep. In this study, a broadly targeted UPLC-MS/MS metabolomics approach and 16s sequencing of gut microorganisms were used to detect, identify and quantify changes in ileal microorganisms, liver metabolites and ileal metabolites following the addition of Chinese herbal preparations. The addition of a 0.5% herbal feed additive increased ileal IgA, IgG and complement C3 levels. The addition of Chinese herbal preparations can increase the abundance of *Firmicutes*, *Actinobacteriota*, *Bacteroidota*, at the portal level of the ileum, increase the metabolism of organic matter and its derivatives, bile acids, amino acids and their metabolites, coenzymes, and vitamins in the liver and ileum, enhance nutrient absorption and waste metabolism, accelerate liver metabolism, promote gut-liver circulation, and improve ileal and liver immunity. This study provides a theoretical basis for understanding the effects of herbal feed additives on the gut-liver axis in ruminants.

## Introduction

In the wake of the complete prohibition of antibiotics, the utilization of Chinese herbal preparations has emerged as a promising alternative in the advancement of animal husbandry. The increasing use of herbal medicines is due in part to the growing problem of antibiotic misuse and resistance. While antibiotics are very effective in treating bacterial infections, prolonged or inappropriate use can lead to the development of bacterial resistance, which reduces the effectiveness of antibiotics, as well as causing adverse effects on livestock (1). In contrast, Chinese herbal medicines are generally considered to have a lower risk of toxicity and drug resistance, and to be of natural origin, versatile, safe, reliable, economical and environmentally friendly. The effectiveness of Chinese herbal medicines in treating certain diseases is comparable to that of antibiotics, and some specific Chinese medicine components have been shown to possess certain antibacterial properties. For example, Atractylodes macrocephala, saponins, Scutellaria baicalensis, and Radix et Rhizoma Ginseng have shown antibacterial, antiviral, anti-inflammatory, and immune-function-enhancing effects (2).With the development of biotechnology, especially the rise of microbiome and metabolomics, more and more studies have shown that intestinal microorganisms and intestinal metabolites can be linked to the organs and tissues of the animal body, which are called “axis”, such as: gut-testis axis, gut-liver axis, and gut-cardiac axis. The gut-liver axis, which governs the nutritional metabolism of animals by means of the interplay between intestinal microecology and the host, plays a crucial role in ensuring the sound growth of animals. The role of gut microbial communities in maintaining host health has attracted widespread attention (3). The liver and intestine are connected through the portal vein, the biliary system, and mediators in the circulation (gut-liver axis). Microorganisms in the gut are involved in the maintenance of liver steady state (4). Nutrients, microbial antigens, metabolites and bile acids regulate metabolic and immune responses in the gut and liver, thereby interacting with each other to influence the structure and function of microbial communities (5). Metabolomics is the science of the study of all metabolites in living organisms, such as amino acids, fatty acids and carbohydrates, to quantitatively analyse and assess changes in metabolite levels and to reveal their relevance to physiological processes. Recently, metabolomics analysis based on ultra performance liquid chromatography-tandem mass spectrometry (UPLC-MS/MS) technology has been widely used in animal physiology and pathology (6). However, the investigation of the gut-liver axis in ruminants remains relatively limited. Consequently, this study primarily employed metabolomics and 16s to examine the impact of Chinese herbal preparations on the intestinal and liver axis of Hu sheep, and This in turn revealed the role of Chinese herbal preparations in regulating nutrient metabolism, gut-liver circulation and immune function in Hu sheep. The group’s previous research shows that: adding 0.5% and 1% of Chinese herbal preparations to concentrate supplements can improve the content of IgM, IgA and lysosomal enzymes in the serum of Hu sheep, and improve the length of small intestinal villi and villus crypt ratio, which indicates that the Chinese herbal preparations can improve the level of immunity of the organism, and significantly promote the development of small intestinal tissues morphology, and improve the absorption and utilization of nutrients. The mechanism of the gut-liver axis is illustrated in **Supplementary Figure S1**. In animals, the intestine and the liver are connected through the portal vein, and about 2/3 of the liver’s blood comes from the intestine, so the liver first receives small-molecule nutrients (e.g., small-molecule proteins, peptides, amino acids, short-chain fatty acids, etc.) absorbed in the intestine. The liver also secretes bile acids into the intestine, most of which (more than 95%) are reabsorbed in the ileum, returned to the liver through the portal vein, and secreted into the bile ducts, forming the enterohepatic circulation. This bi-directional enterohepatic connection is a bridge for communication in the gut-intestinal microbes-liver axis, which plays an important role in regulating nutrient metabolism and immunity in animals. Gut microbes regulate animal growth and metabolism through signals (e.g., short-chain fatty acids, bile acids, methylamine, amino acid derivatives, and microbe-associated molecular pattern MAMP), which are sensed by the host through T-cell-like receptors, free fatty acid receptors, and bile acid receptors to regulate nutritional metabolism and immunity.

## Materials and methods

### Ethics statement

The animal committee of Gansu Agricultural University approved all animal management and experiments (ethical approval number: GSAU-AEW -2020-0057).

### Chinese herbal preparations

Chinese herbal preparations are mainly composed of *Codonopsis pilosulae*, *Medicated leaven*, *Malt*, *Hawthorn* (Fried), *Astragalus membranaceus*, *Poria cocos*, *Atractylodes*, etc. The colorimetric method was used to determine the effective content of Chinese herbal preparations, and they were made into bulk formulations, which were fed to Hu sheep according to the ratios of concentrate supplement 0.5% and 1% respectively. We showed that the three established methods for the determination of the effective active ingredients were reliable by examining the linear relationship, precision, stability, reproducibility and spiking recovery experiments: the contents of total polysaccharides, total flavonoids and total saponins of the herbal preparations were 27.21%, 0.03% and 0.12%, respectively, of which total polysaccharides had the highest content. Nutritional levels and active ingredient contents of Chinese herbal preparations are shown in **Table 1**, and diet composition and nutrient content are shown in **Table 2**.

**Table 1:** Nutritional content of Chinese herbal preparations.

Items	Content/%
Dry matter	6.22
Ash	11.17
CP	11.84
Neutral detergent fiber	23.69
Acid detergent fiber	11.26
Calcium	0.97
Phosphorus	1.94

Ash, crude ash; CP, crude protein.

**Table 2:** Composition and nutrient level of basal diet (as-fed basis).

Ingredients	Content/%
Ingredients	
Corn	40
Cottonseed meal	5.5
Soybean meal	12.5
Peanut seedling	29
Wheat bran	9
CaHPO4	0.5
NaCl	1
Limestone	1.5
Premix ^1^	1
Total	100
Nutrient levels^2^	
ME(MJ/kg)	8.57
CP	14.51
EE	2.21
NDF	30.11
ADF	19.58
Ca	1.01
P	0.47

^1^ The premix provided the following per kg of diets: Fe (as ferrous sulfate) 75 mg, Cu (as copper sulfate) 14 mg, Zn (as zinc sulfate) 80 mg, Se (as sodium selenite) 0.25 mg, I (as potassium iodide) 0.25 mg, Co (as cobalt) 0.30 mg, Mn (as manganese sulfate) 400 mg, VA 3 000 IU, VD 500 IU, VE 200 IU.

^2^ ME was a calculated value, while the others were measured values.

### Test animals

The trial was initiated from March to July 2023 Gansu Province, Wuwei City, Gulang County. With an average initial body weight of (19.57 ± 1.56 kg) of 18 healthy male Hu sheep were selected as test animals. Randomly divided into 3 groups, each group of 6 animals, they were control group (Con) (fed basic ration), test I (T1) (fed 0.5% Chinese herbal preparation of concentrate in the ration), test II (T2) (fed 1% Chinese herbal preparation of concentrate in the ration). The pre-test period was 7d (The trial was conducted under the same housing, lighting, and ventilation conditions, with the sheep fed ad libitum in a loose feeding system. A 10-day acclimation period was followed by a 90-day experimental period, with feeding starting at 7:30 am each day. The concentrated supplement was mixed with the feed for the first 10 days, and then gradually increased in proportion to the concentrated supplement containing 0.5% and 1% of the herbal preparation. Specifically, 1/4 of the concentrated supplement was added on days 1-2, 1/3 on days 3-4, 2/3 on days 5-6, and the full amount on day 7. During the trial, the sheep had free access to water to ensure that the feeding method was consistent across all groups), and formal experiment was 90 d. To study the metabolic changes of Chinese herbal preparations on the gut-liver axis of Hu sheep, we performed extensive metabolic analyses of ileum and liver axis metabolites by using a broadly targeted UPLC-MS/MS metabolomics approach and 16S sequencing of ileal microorganisms.

### Sample preparation

Liver tissue samples, ileal contents samples were collected from the test sheep rapidly after slaughtering, immediately place in a tank of liquid nitrogen and return to the laboratory within 1 h. Store in a refrigerator at -80°C for total RNA and protein extraction, and another portion of the sample was fixed in 4% paraformaldehyde solution. Then the gradient alcohol dehydration, xylene transparency, paraffin embedding, serial sectioning, 4 μm thick. Liver and gut samples were removed from the -80°C refrigerator. The samples were thawed on ice and the thawed samples were chopped and mixed. Each sample was accurately weighed 20 mg using multipoint sampling and then transferred to a centrifuge tube and homogenized with a steel ball (30Hz, 20s). Following centrifugation (3000 rpm, 4°C, 30 s), the pellet was added to 400 mL of 70% methanol-water internal standard extractant and shaken well (1500 rpm) for 5 min. It was then centrifuged (12,000 rpm, 4°C, 10 min). To collect the supernatant for analysis, transfer 300 mL of supernatant to a new centrifuge tube, allow to stand for 30 minutes at 20°C, then centrifuge (12,000 rpm, 4°C) for 3 minutes.

### Ileal immunity indexes

Ileal immunity indexes included immunoglobulin A (IgA), immunoglobulin G (IgG), immunoglobulin M (IgM), complement 3 (C3) and complement 4 (C4) content. The above indicators were determined by using kits, which were purchased from Beijing Huaying Biotechnology Co.

### Chromatography mass spectrometry acquisition conditions

The data acquisition instrument system mainly includes ultra-high performance liquid chromatography (Ultra Performance Liquid Chromatography, UPLC) (ExionLC AD, https://sciex.com.cn/) and tandem mass spectrometry (Tandem mass spectrometry, MS/MS)(OTRAP^®^, https://sciex.com.cn/) T3 Method Chromatographic Acquisition Conditions: Chromatographic column: Waters ACQUITY UPLC HSS T3 C18 1.8 um, 2.1 mm*100 mm; Mobile phase: Ultrapure water (0.1% formic acid) in phase A, and acetonitrile (0.1% formic acid) in phase B. Elution gradient:0 min water/acetonitrile (95:5 V/V), 11.0 min at 10:90 V/,12.0 min at 10:90 V/, 12.1 min. Flow rates 0.4 ml/min; column temperature 40°C; injection volume 2 ml. Mass Spectrometry Acquisition Conditions: Electrospray ionization (ESI) temperature 500°C, mass spectrometry voltage 5500 V (positive), -4500 V negative), ion iS I) 55 psi, gas II (GS H) 60 psi, curtain gas (CUR) 25 psi, touch ted dissociation, CAD) parameter was set to high. In a triple quadrupole (Qtrap), each collision-induced ionization (colli ion pair was detected by scanning based on the optimized jade (declustering potential, DP) and collision energy (CE).

### Metabolomics analysis raw

Raw UPLC-MS/MS data were imported into Analyst 1.6.3 software. Metabolites were analyzed qualitatively and quantitatively for each sample using mass spectrometry (MS) based on local metabolite databases. Data were statistically analyzed by log2 transformation to improve normality and standardization. Hierarchical cluster analysis (HCA) and orthogonal partial least squares discriminant analysis (OPLS-DA) were performed using R software. Principal Component Analysis (PCA) was used to simplify and downscale high dimensional complex data. According to OPLS-DA analysis, the criteria for screening differential metabolites were variables with a fold change (FC) ≥2 or ≤0.5 and VIP ≥1. Relationships between differential metabolites were demonstrated by Venn diagrams. Metabolite annotation and pathway enrichment analyses were performed using the KEGG Compound Database and KEGG Pathway Database.

### Intestinal flora analysis

Total DNA was extracted from microbial samples using the kit.DNA quality was measured by 0.8% agarose gel electrophoresisDNA was quantified by UV spectrophotometer.Specific regions of the 16S rRNA gene were amplified by PCR using specific universal primers.The 16S rRNA gene is present in the genomes of all bacteria and is highly conserved and has specific variable regions. The PCR products were purified and libraries were constructed for sequencing. Sequencing was performed using the Illumina MiSeq high-throughput sequencing platform. Process and analyze sequencing data, including sequence quality control, OTU clustering, species annotation, and diversity analysis. Common methods for data analysis included principal coordinate analysis (PCoA), non-metric multidimensional scaling analysis (NMDS), linear discriminant analysis (LEfSe), etc. In this test microbial composition analysis, we focused on the top 10 bacteria at the phylum and genus level. We analyzed the microbial composition at each taxonomic level. The experimental data were analyzed by one-way analysis of variance (ANOVA) using the ANOVA procedure of the SPSS 26.0 statistical software, and Duncan’s method for multiple comparison test, and the results were expressed as mean ± standard deviation, with *P*<0.05 indicating significant differences. Statistical analyses and graphing of differential genera at the ileal midgut level and genus level were performed using Graphpad 5.0 Prism software.

## Results

### Liver tissue section results

**Figure 1:**
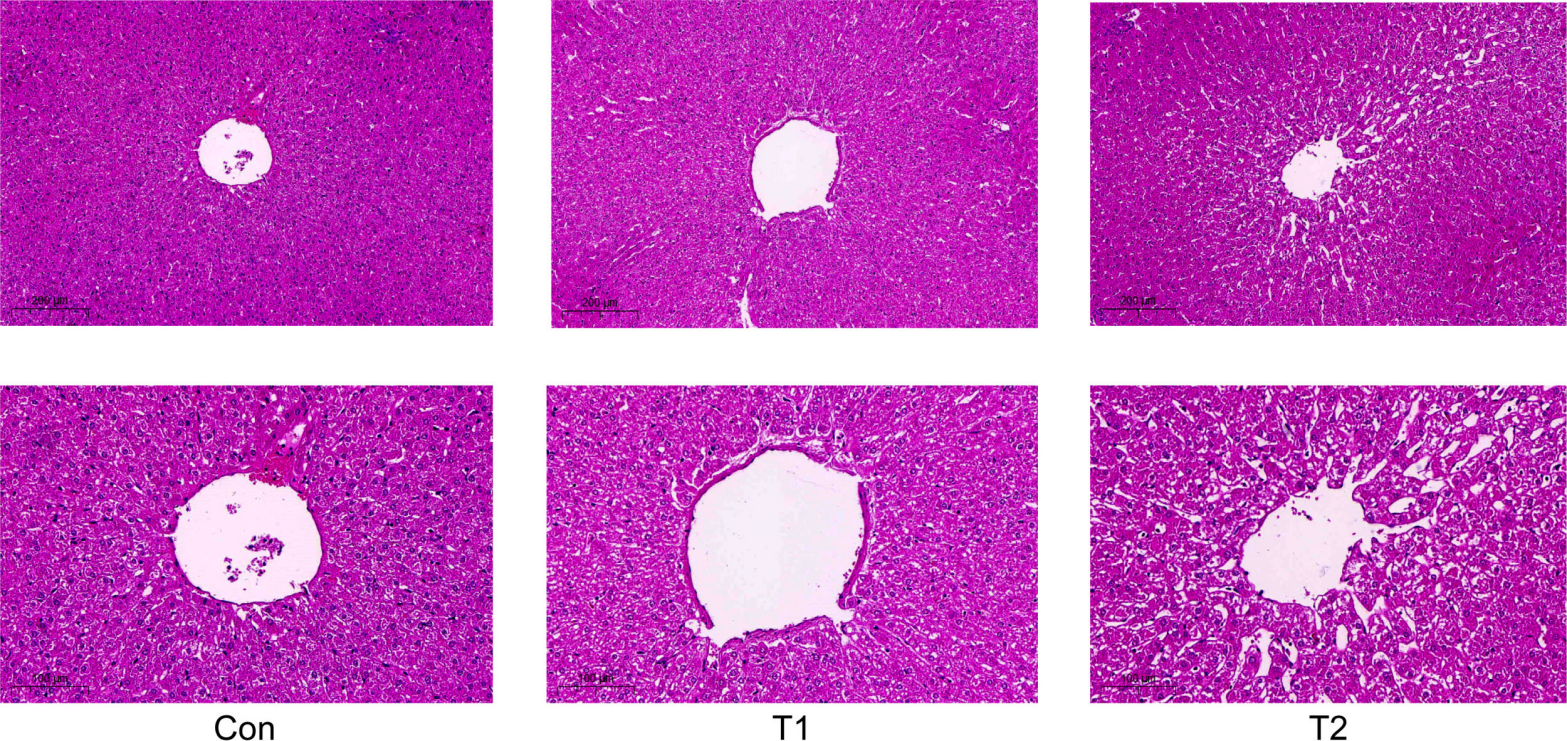
Effect of adding herbal feed additives to the ration on the liver tissue section of Hu Sheep.

After feeding the herbal feed additives to the Hu sheep, it was observed through liver tissue sections, as shown in (**Figure 1**), the liver morphology of the Hu sheep in the control group was more regular, and the liver cord was neatly arranged and a small amount of collagen fibers could be seen in the portal area of the liver tissue, while there was hyperplasia of collagen fibers in the portal area of the liver tissue of the Hu sheep in the T1 and T2 groups, and the formation of fibrous intervals could be seen, but the difference was not obvious. Compared with the control group, T1 and T2 lymphocytes increased but the difference was not obvious, and there was some hyperplasia in connective tissues but the difference was not obvious, which indicated that the herbal preparation had no obvious lesions and abnormalities on the liver of the Hu sheep, and indicated that the herbal feed additives could be used for the healthy reproduction of the Hu sheep, and the side effects on the liver tissues of the Hu sheep were small.

### Effects of herbal feed additives on ileal immunity indexes of Hu sheep

As can be seen from **Table 3**, the serum IgA, IgG, and complement C3 contents of the test I group were significantly higher than those of the control group (*P*<0.05); there was no significant difference in serum C4 and IgM contents between the groups (*P*>0.05).

**Table 3:** Effects of herbal feed additives in diets on ileal immunity indexes in Hu sheep.

Items	Groups			*P*-value
	Control	Test Group I	Test Group II	
IgG g/L	0.42 ± 0.05^b^	0.56 ± 0.01^a^	0.44 ± 0.04^b^	0.002
IgA g/L	0.08 ± 0.02^b^	0.21 ± 0.01^a^	0.09 ± 0.04^b^	0.001
IgM g/L	0.02 ± 0.01^b^	0.03 ± 0.01^a^	0.02 ± 0.01^b^	0.001
Complement 3 g/L	0.02 ± 0.03^b^	0.03 ± 0.01^a^	0.02 ± 0.01^b^	0.049
Complement 4 g/L	0.01 ± 0.01	0.01 ± 0.01	0.01 ± 0.01	0.070

The three groups were compared; same letters were not significant, different letters were significant.

### UPLC-MS/MS-based qualitative and quantitative analyses of metabolites

To investigate the metabolic alterations occurring in the gut-liver axis under varying proportions of Chinese herbal preparations, we conducted metabolome sequencing of the ileum. The ileum was specifically identified and analyzed, resulting in the detection of a total of 1202 metabolites. These metabolites were further classified into various categories, including 378 amino acids and their metabolites, 102 Benzene and its derivatives, 51 alcohols and amines, 21 bile acids, 21 coenzymes and vitamins, 94 glycerophospholipids, 87 nucleotides and their metabolites, 31 hormones and hormone-related substances, and 22 aldehydes ketones and esters, 51 carbohydrates and their metabolites, 164 organic acids and their derivatives, 71 heterocyclic compounds, and 89 fatty acyls. The main components of Hu sheep ileums are amino acids and their metabolites and organic acids and their derivatives.

### Different ratios of Chinese herbal preparations on ileal metabolite differences

**Figure 2:**
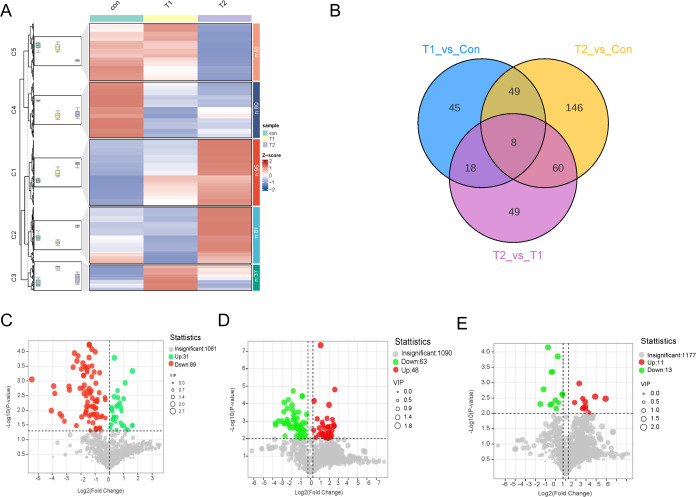
Differences metabolite accumulation in ileal between different proportions of Chinese herbal preparations. **(A)** Trends of metabolite changes in ileal clustering profiles. **(B)** Venn diagrams of metabolite differences between Con vs T1, Con vs T2, and T1 vs T2. **(C-E)** Volcano plots of metabolite differences between Con vs T1, Con vs T2, and T1 vs T2.

OPLS-DA assessed the explanatory power and predictive ability of the model by R^2^Y and Q^2^. In this study, OPLS-DA was used to analyse the differences in ileal metabolites between different groups and to examine the effect of Chinese herbal preparations, and significant metabolite differences and good group differentiation were found. OPLS-DA was used for Con vs. T1, T1 vs. T2, and Con vs. T2. the R^2^Y values were 0.99, 0.997, and 0.971, respectively (**Supplementary Figure S2D-F**). It suggested that the OPLS-DA model is well fitted. Highly predictable and suitable for subsequent data analysis. According to the OPLS-DA results, groups of differential metabolites were further selected and classified. Further, we used FC ≥ 1.2 to select the results, which are represented by Volcano, Venn, heat and k-means diagram. To study the significant changes in the ileum metabolites of different proportions of Chinese herbal preparations, we analyzed the different metabolites between Con vs T1, T1 vs T2, Con vs T2. Cluster graphs and heat maps show clear clustering of metabolite variants, and the trend of variability is shown in **Figure 2A**. Based on the Vennen diagrams for the different groups in **Figure 2B**, a total of eight different metabolites were simultaneously identified in the three groups. For example, organic acids and their derivatives, benzene and their derivatives, nucleotides and their metabolites, bile acids, amino acids and their metabolites, hormones and hormone-related substances were identified as overlapping differential metabolites (**Supplementary Table S1**). for example: 2-Hydroxyphenylacetic acid is an organic compound with antioxidant properties, which can act on β2 receptors on intestinal smooth muscle cell. beta-Muricholic acid is a bile acid that also plays an important role in the gut, it can promote the production and removal of bile. It can stimulate the secretion and excretion of bile by acting on G protein-coupled receptors on the surface of hepatocytes, thus maintaining the normal circulation of bile. L-lysine-L-alanine is one of the essential components of glutathione synthesis. Involved in glutathione synthesis: Glutathione is the main intracellular antioxidant, able to scavenge free radicals and peroxides also acts as a metabolic substrate for intestinal microorganisms, influencing the composition and function of the intestinal microbiota, which in turn influences the antioxidant capacity of the intestines. 6-Methylaminopurine is an antimetabolite. It also has a role in the intestinal tract, which can inhibit the growth and multiplication of bacteria in the gut. A total of 120 metabolites with significant differences were identified in Con vs T1 and included 31 up-regulated and 89 down-regulated metabolites (**Figure 2C**). A total of 263 metabolites with significant differences were identified in Con vs T2 (**Figure 2D**). Includes 134 up-regulated and 129 down-regulated metabolites. Among the differential metabolites between Con vs T2 and T1 vs T2 exhibited numerous up-regulated metabolite. This result suggested that a large number of metabolites accumulated in the ileum after the addition of Chinese herbal preparations. Most of them are up-regulated. T1 vs T2 identified a total of 135 metabolites with significant differences and included 91 up-regulated and 44 down-regulated metabolites (**Figure 2E**).

### Metabolic profile of metabolites in the ileum by different proportions of Chinese herbal preparations

To further investigate the changing patterns of differential metabolite. In the ileum supplemented with different proportions of Chinese herbal preparations. The six classes of differential metabolites were analyzed by heat mapping (**Supplementary Figure S3**). The results showed a decreasing trend in most bile acids, organic acids and their derivatives, heterocyclic compounds hormones and their related substances, and an increasing trend in amino acids and their metabolites, and carbohydrates.

### Metabolic pathways of ileal metabolites by different ratios of Chinese herbal preparations

**Figure 3:**
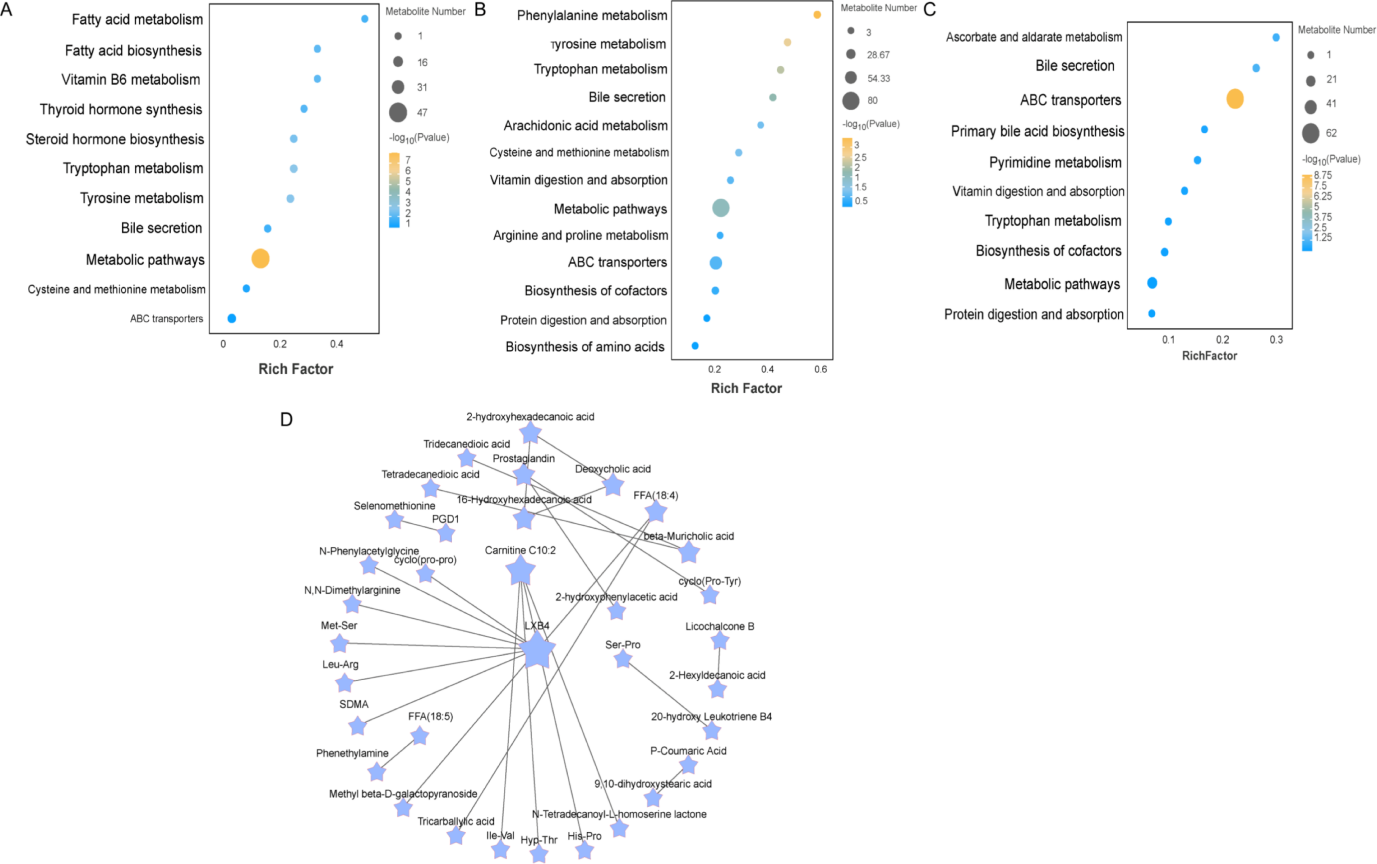
Enrichment analysis of differentially expressed metabolite pathways and related network diagrams. **(A-C)** KEGG pathway enrichment analysis of differentially expressed metabolites of Con vs T1 and Con vs T2 and T1 vs T2. The color of the dots represents the p-value and the size of the dots represents the number of differentially expressed metabolites. **(D)** KEGG pathway-based intergroup differential metabolite interaction diagram.

To explore the potential metabolic pathways of the ileum in different proportions of Chinese herbal preparations. According to the differential metabolites generating KEGG classification and bubble diagrams as in (**Figure 3**), Identify potential developmentally relevant biomarkers (to gut-liver axis nutrient metabolism and antioxidant and immunity). Differential metabolites were screened and annotated, and more than five metabolites involved in the KEGG pathway were selected and subjected to cluster analyses to investigate changes in metabolic patterns at different ratios. The Con and T1(**Figure 3A**) subgroups differed in steroid hormone synthesis, as well as in the pathways of tryptophan, phenylalanine, and tyrosine metabolism, bile secretion, vitamin digestion and absorption, amino acid and nucleoside glycoside metabolism, and glutathione and arachidonic acid metabolism. The Con and T2 (**Figure 3B**) subgroups differed in the metabolic pathways of metabolites, mainly the metabolism of phenylalanine and tryptophan, steroid biosynthesis, secretion of bile acids, metabolism of arachidonic acid, secretion of primary bile acids, digestion and absorption of vitamins. The T1 and T2 subgroups (**Figure 3C**) were predominant in the metabolic pathways of metabolites: steroid metabolism, bile acid and primary bile acid secretion, cholesterol metabolism, vitamin digestion and absorption, and arachidonic acid metabolism. Following this, pathway interactions were analyzed for the three groups of differential metabolites in the ileum. We selected the top 25 differential metabolites to map the interactions (**Figure 3D**). We found that fatty acyl metabolites (LXB4, Carnitine C10:2) were closely associated with amino acids and their metabolites Ile-Val, cyclo (pro-pro), the Organic acids and their derivatives (SDMA). beta-Muricholic acid was closely associated with Tridecanedioic acid, Tetradecanedioic acid. These metabolites synergize with each other to promote the intestinal barrier of the ileum and the absorption and metabolism of nutrients.

### Qualitative and quantitative metabolite analysis based on UPLS-MS

To study the metabolic changes in the liver of different proportions of herbal preparations. We performed liver metabolome sequencing and identified and analyzed a total of 1059 metabolites in the liver, classifying the various metabolites in the liver into different categories. Among them, 333 were amino acids and their metabolites, 81 were benzene and its derivatives, 45 were alcohols and amines, 12 were bile acids, 15 were coenzymes and vitamins, 95 were glycerophospholipids and glycerolipids, 77 were nucleotides and their metabolites, 20 were hormones and hormone-related substances, 18 were aldehydes, ketones and esters, 4 were tryptophan, cholinergic and pigmentary substances, 52 were carbohydrates and their metabolites, 52 were organic acids and its derivatives 155 kinds, 62 kinds of heterocyclic compounds and 76 kinds of fatty acyl groups. The main components of goat liver include amino acids and their metabolites, organic acids and their derivatives.

### Differences in liver metabolites by different proportions of Chinese herbal preparations

**Figure 4:**
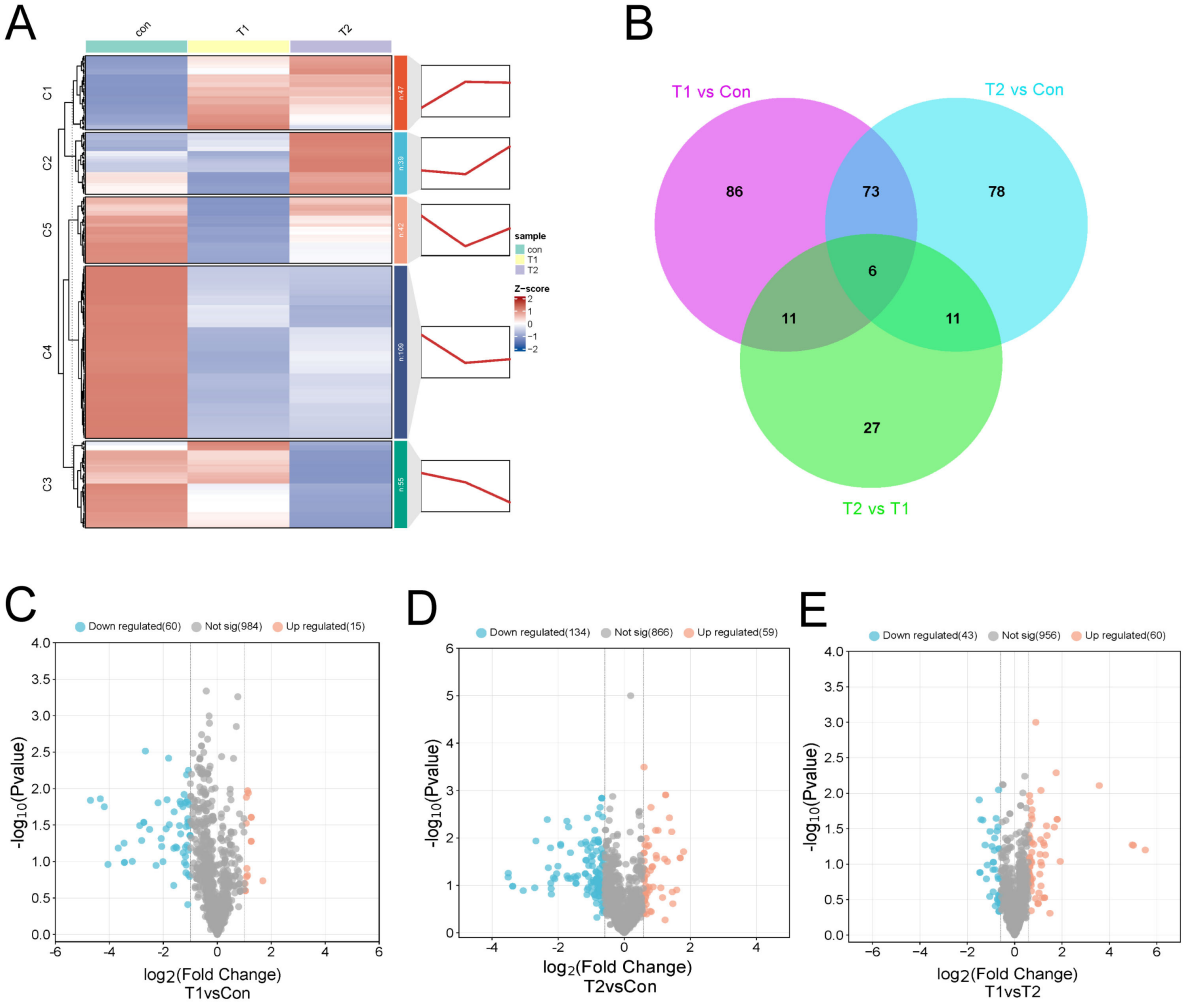
Differences in liver metabolite between different proportions of Chinese herbal preparations. **(A)** Trends of metabolite changes in liver clustering profiles. **(B)** Venn diagrams of metabolite differences between Con and T1, Con vs T2, and test I vs T2. **(C-E)** Volcano plots of metabolite differences between Con vs T1, Con vs T1, and T1 vs T2.

The OPLS-DA analysis revealed R^2^Y values of 0.998, 0.998, and 0.998 for the comparisons of Con vs T1, T1 vs T2, and Con vs T2, respectively (**Supplementary Figure S4D-F**), indicating a well-fitted, predictable, and appropriate OPLS-DA model for subsequent data analysis. according to the OPLS-DA results, differential metabolites were further screened and classified. Further, we used FC ≥ 1.2 to select the results represented by Volcano, Venn, heat and k-means diagram. In order to investigate the significant changes in liver metabolites of different ratios of Chinese herbal preparations, we analyzed the differential metabolites between Con vs T1, T1 vs T2, and Con vs T2. A total of 176 metabolites with significant differences were identified in Con vs T1, including 30 up-regulated and 146 down-regulated metabolites. A total of 55 metabolites with significant differences were identified for T1 vs T2. This included 35 up-regulated and 20 down-regulated metabolites. Con vs T2 identified a total of 168 metabolites with significant differences. Includes 55 up-regulated and 113 down-regulated metabolites. Among the differential metabolites between T1 vs T2 showed many up-regulated metabolites. This result suggested that a significant numerous metabolites accumulated in the ileum after the addition of Chinese herbal preparations. Most of them are upwardly mobile. The thermograms of the differential metabolites between the three combinations also clearly show the trends described above. Cluster plots and heat maps showed clear clustering of metabolite variation, and the trend of variation is shown in (**Figure 4**). Based on the Wayne diagrams of the different groups. A total of 191 differential metabolites were identified confirming the dynamic changes in liver metabolites by the addition of herbal preparations. Also, six differential metabolites such as organic acids and their derivatives, heterocyclic compounds, bile acids, amino acids and their metabolites were identified as overlapping differential metabolites.

### Characteristics of metabolism of metabolites in the liver by different proportions of Chinese herbal preparations

To further investigate the changing pattern of differential metabolites, heat map analysis of eight types of differential metabolites was carried out in livers supplemented with different proportions of Chinese herbal preparations (**Supplementary Figure S5**). The results showed that most of the amino acids and their metabolites, benzene and their derivatives, bile acids, organic acids and their derivatives, carbohydrates and their related substances showed a decreasing trend.

### Metabolic pathways of liver metabolites in different proportions of Chinese herbal preparations

**Figure 5:**
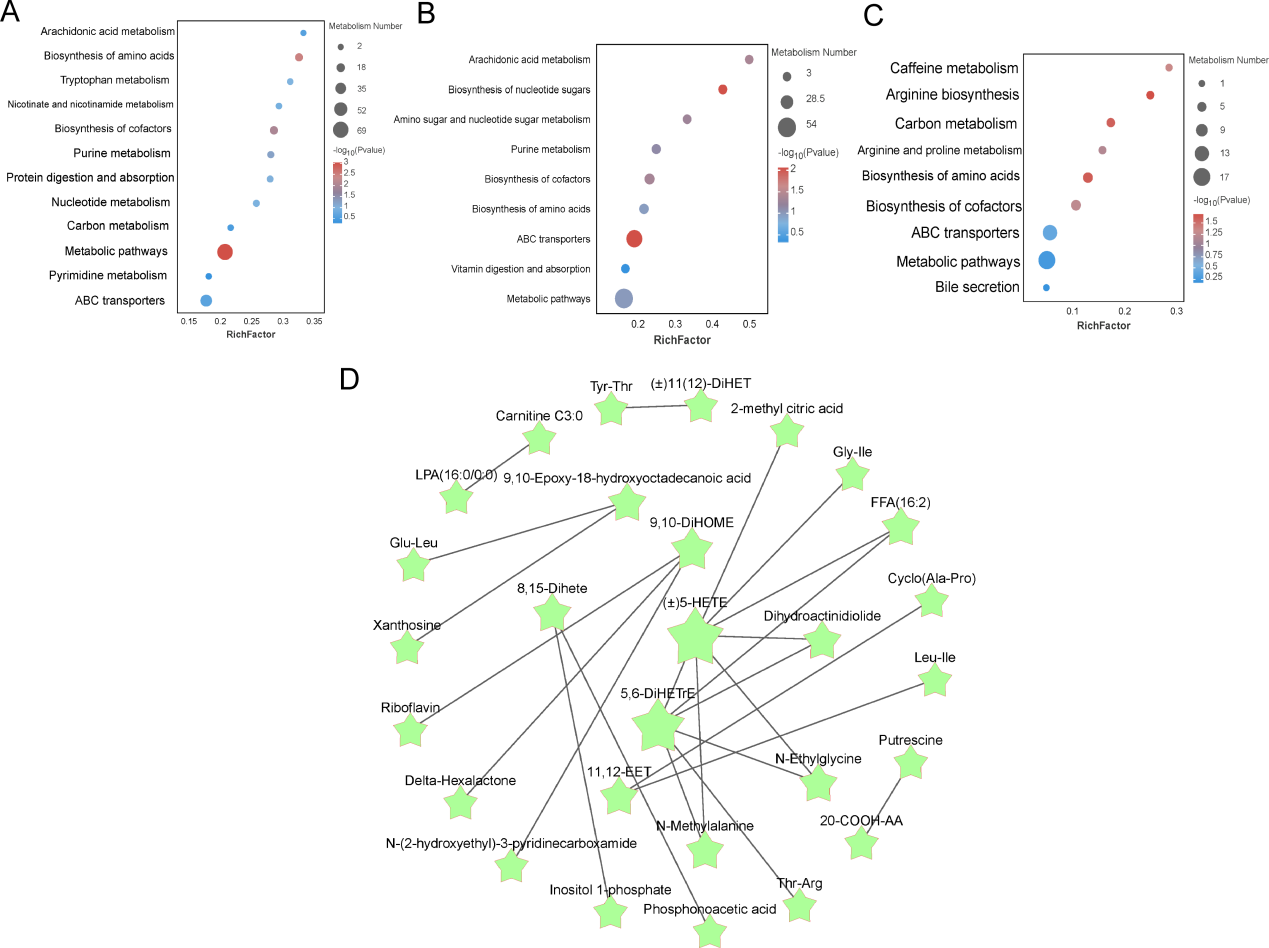
Enrichment analysis of differentially expressed metabolite pathways in liver and related network diagrams. **(A-C)** KEGG pathway enrichment analysis of differentially expressed metabolites in Con vs T1, Con vs T2 and T1 vs T2. The color of the dots represents the p-value and the size of the dots represents the number of differentially expressed metabolites. **(D)** KEGG pathway-based interaction plot of differentially metabolites between groups.

Differential metabolites identified based on screening criteria were annotated using KEGG annotation information. In order to better investigate the different metabolic patterns of potential metabolic pathways in different proportions of livers (**Figure 5**). the major metabolic pathways for the Con and T1 subgroups of differential metabolites were: arachidonic acid metabolism, vitamin digestion and absorption, glutathione metabolism, primary bile acid biosynthesis, and bile secretion. The main metabolic pathways for the Con and T2 subgroups of metabolites were: arachidonic acid metabolism, glycolysis, bile secretion, vitamin digestion and absorption, cholesterol metabolism, glutathione metabolism, protein digestion and absorption, vitamin B6 metabolism, primary bile acid biosynthesis. The major pathways of T1 and T2 include glycolysis, bile secretion, glutathione metabolism, retrograde endogenous cannabinoid signaling, protein digestion and uptake, and vitamin digestion and uptake. Meanwhile, the major metabolic pathways associated with the differential metabolites mainly involve energy metabolism, bile acid metabolism with gut-liver axis immunity, circulation and antioxidant effects. L-isoleucine is one of the important raw materials in the liver, which promotes the synthesis of albumin, globulin and other important proteins by liver cells. Subsequently, the selected relevant metabolites were analyzed for pathway interactions. The differential metabolite pathways of the interacting mapped Con vs T1 groups, Con vs T2 groups, and T1 vs T2 groups are shown in **Figures 5A-C**. Subsequently, pathway interactions were analyzed for the three groups of differential metabolites. We selected the top 25 differential metabolites and plotted the interactions **Figure 5D**. We found that the fatty acyl metabolites ((±)5-HETE), 5,6-DiHETrE were located in the central of the graph, and were closely associated with other metabolites such as the amino acid and its derivatives (N-Methylalanine) Gly-Ile, aldehydes and ketones metabolites (N-Ethylglycine), and organic acids and their metabolites (2-methyl citric acid) are closely related. Together, these metabolites promote liver antioxidant, anti-inflammatory, and nutrient uptake and substance metabolism.

### Ileum microorganisms

**Figure 6:**
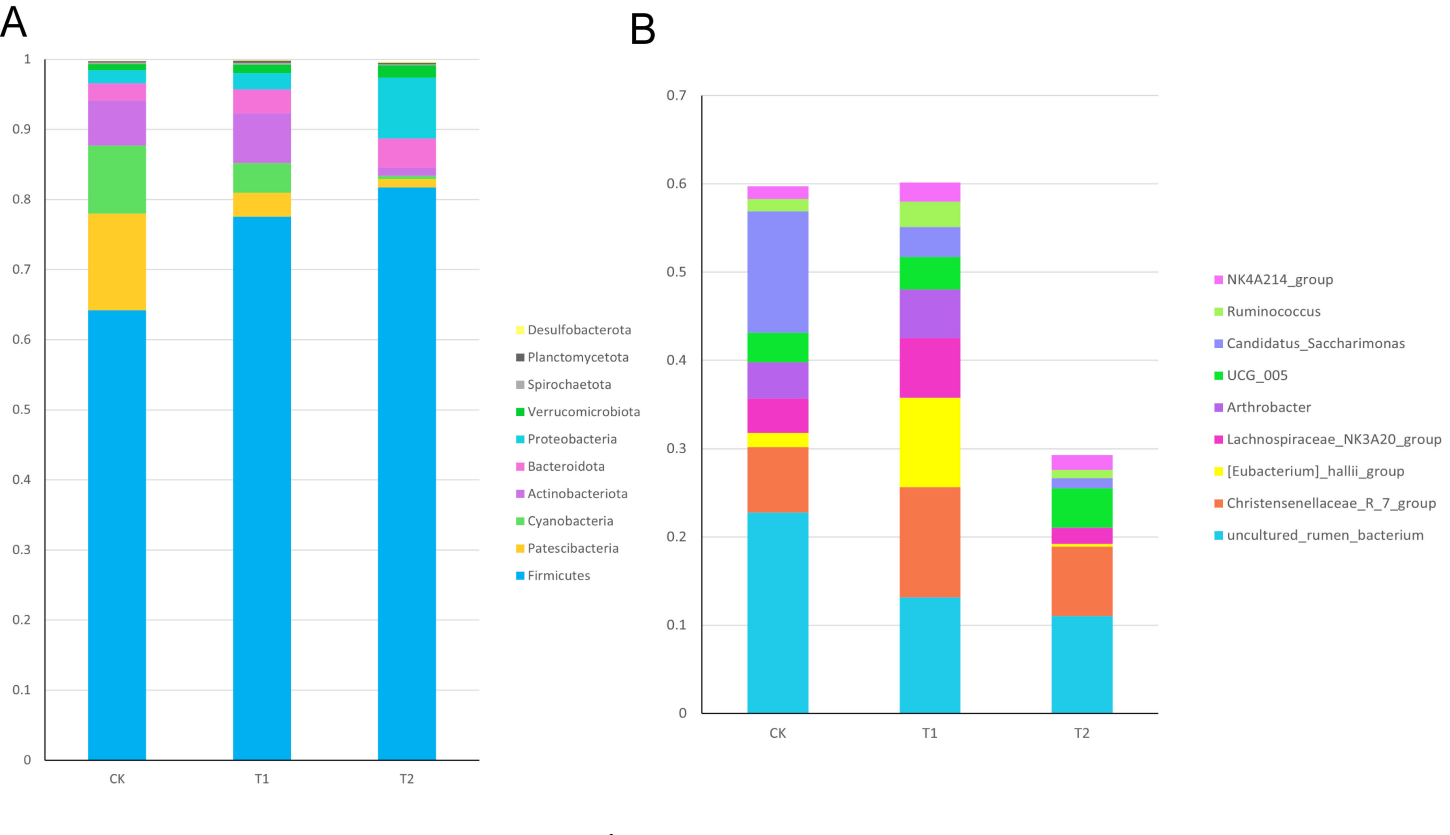
Relative abundance of ileal bacterial in Hu sheep **(A)** Relative abundance of ileal bacterial phylum levels in Hu sheep **(B)** Relative abundance of ileum bacterial genus levels in Hu sheep.

To further investigate whether Chinese herbal preparations affect the intestinal flora, we analyzed the composition of the ileal flora by 16srRNA gene sequencing. The structure of microbial composition at the phylum level and genus level is shown in (**Figure 6**). At the phylum level, there was an increasing trend in the phylum of *Firmicutes*, *Actinobacteriota*, *Bacteroidota*, *Proteobacteria*, *Verrucomicrobiota* compared to the Con. At the genus level, there was an increasing trend in the *Christensen’s algae R_7_group*, *Eubacterium hallii_group*, *Lachnospiraceae_NK3A20_group*, *Arthrobacter*, and *NK4A214_group* compared to the Con.

### Correlation analysis

**Figure 7:**
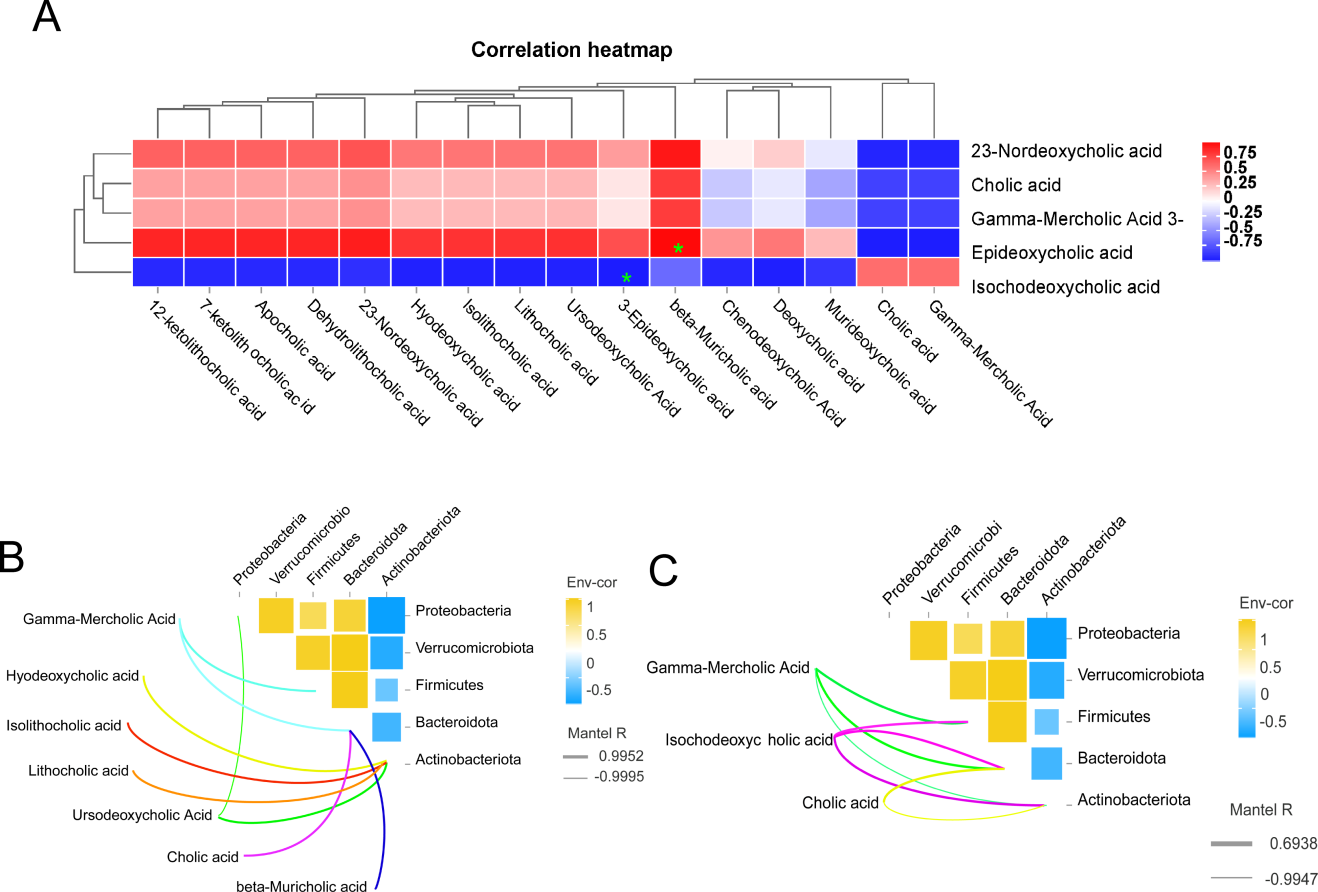
Correlation analysis. **(A)** correlation analysis of bile acid in ileal and liver, **(B)** Correlation analysis of ileal microbes and ileal bile acids, **(C)** Correlation analysis of ileal microbes and liver bile acids.

Bile acids (BAs) are the final byproducts of liver cholesterol metabolism, synthesized within the liver, and subsequently transported as precursor-bound bile acids across the tubular membranes of hepatocytes to the biliary system, which ultimately eliminates bile acids into the small intestine (7). The heat map analysis of the correlation between ileum bile acids and liver bile acids is shown in (**Figure 7A**). For example, 3-epideoxycholic acid in the ileum was significantly negatively correlated with isodeoxycholic acid in the liver (*P*<0.05). There was a significant positive correlation (*P*<0.05) between β-deursolic acid in the ileum and 3-epi-deoxycholic acid in the liver. Based on the findings presented in (**Figure 7B**), it was observed that Gamma-Mercholic Acid exhibited a positive correlation with the thick-walled phylum in the ileum. Furthermore, Gamma-Mercholic Acid and Cholic acid, beta-Muricholic acid were identified as relevant bile acids for Bacteroidota. Additionally, Hyodeoxycholic acid, Isolithocholic acid, Lithocholic acid, Ursodeoxycholic Acid were found to be positively correlated with Actinobacteriota. According to (**Figure 7C**), we found that the bile acids in the liver that correlated with *Firmicute*s were Gamma-Mercholic Acid, Isochodeoxycholic acid, and showed a positive correlation. The bile acids of relevance to the *Bacteroidota* are Gamma-Mercholic Acid, Cholic acid and Isochodeoxycholic acid. The bile acids correlated with *Actinobacteriota* were Gamma-Mercholic Acid, Cholic acid and Isochodeoxycholic acid, with a positive trend.

## Discussion

The ileum plays a crucial role in the gut-liver axis in ruminants, specifically in the absorption of amino acids. Short chain fatty acids (SCFAs) serve as crucial metabolites produced by gut microorganisms. Several studies have demonstrated the significant role of short-chain fatty acids (SCFAs) in the maintenance of host health and disease. Primarily, short-chain fatty acids (SCFAs) have the ability to regulate the immune system and enhance the body’s resistance to pathogenic microorganisms. Additionally, SCFAs can promote the absorption of nutrients in the small intestine, particularly for fat and protein (8). SCFAs, such as butyric acid and propionic acid, serve as a source of nutrients and energy for the intestinal epithelium. Propionic acid also acts as a precursor for lipogenesis and gluconeogenesis. Moreover, maintaining appropriate levels of butyric acid in the gut is crucial for preserving intestinal integrity and permeability (9). Several studies have demonstrated that the dietary addition of astragalus polysaccharides significantly increases the levels of acetic acid, propionic acid, butyric acid, taurine, and bile acids in the ileum, thereby regulating intestinal immune function (10). In this experiment, the addition of 0.5% Chinese herbal preparations increased the short-chain fatty acid content (propionic acid and butyric acid) in the ileum, which could enhance the immunity of the ileum. Bile, an essential liver secretion. Bile acids, which are the primary organic solute components of bile, are synthesized from cholesterol in the liver and stored in the gallbladder. Following intestinal secretion, bile acids are reabsorbed in the distal small ileum (11). Cholic acid (CA) and goose deoxycholic acid (CDCA) are produced from cholesterol in the liver through a series of enzymatic reactions, combining with glycine to form bile acids, which are then released into the bile. BAs are then secreted through the gallbladder into the intestine (12). Several studies have demonstrated that bile acids enhance nutrient absorption and regulate the innate immune system of the gut and liver (13). Bile-removing acids are important in the ileum, it not only promotes fat digestion and absorption, but also has various physiological functions such as regulating cholesterol metabolism and maintaining intestinal microecological balance. Depurative bile acids significantly increased the abundance of probiotic species. For example, Bifidobacterium bifidum, which promotes lipid metabolism via the fatty acid-liver peroxisome proliferator-activated receptor alpha (PPAR-α) signaling pathway, which in turn upregulates liver FXR (13). The study demonstrated that the administration of CDCA supplementation in mice effectively restored the LPS-induced intestinal permeability and mitigated the damage to the intestinal barrier (14). Our results showed that levels of pro-goose deoxycholic acid, deoxycholic acid, γ-rat bile acid, rat deoxycholic acid and 1-aziridinyl ethanol were significantly higher in T1 compared to Con and T2, suggesting that the addition of 0.5% of the Chinese herbal preparation to the diets of Hu sheep enhances the assimilation of bile acids and nutrients in the ileum, as well as modulating the immune system. The metabolites derived from tryptophan, including indole-3-acetic acid, indole-3-aldehyde, and indole-3-acrylic acid, contribute to the maintenance of intestinal immune homeostasis and exert a positive influence on both adaptive and innate immune responses in the ileum. These findings highlight the beneficial impact of these tryptophan metabolites on the host’s immune system (15). Indoles, such as 5-hydroxyindole pyruvic acid, have been identified as potential biomarkers for diarrhea. In animal studies, the supplementation of Ginseng and Atractylodis Macrocephalae in the diet has been shown to enhance the microenvironment of intestinal flora and prevent diarrhoea (16). In the present study, the addition of Chinese herbal preparations to the ration resulted in increased levels of 5-hydroxyindole pyruvate in the ileum of Hu sheep, which contributed to help to prevent Hu sheep diarrhoea. Additionally, Indole-3-lactic acid has been found to improve intestinal epithelial cell damage, promote the proliferation of intestinal stem cells, and mitigate oxidative stress in the intestinal epithelium (17). In this experimental study, the levels of indole-3-lactic acid were found to be significantly higher in the test group compared to the control group, suggesting that the herbal preparation contributed to the enhancement of the ileum’s barrier function in Hu sheep.

The key to the gut-liver axis is in the liver, the animal intestine is connected to the liver via the portal vein, and 2/3 of the liver blood comes from the intestine. Intestinal microbiota is the axis that affects gut-liver physiopathology (18). SCFAs are fermentation products of intestinal microorganisms, and high concentrations of SCFAs affect immunity through the intestine and portal vein, and have a significant effect on gut-liver immunity. Propionic acid is a precursor of hepatic gluconeogenesis, butyric acid is partially used for hepatic ATP metabolism, and butyric acid activates gluconeogenesis. SCFA regulate gut metabolic proteins (19). For example, Beta-alanine also has an antioxidant effect, which reduces oxidative stress caused by free radicals and fights against cell and tissue damage. β-alanine is a precursor of coenzyme A, which promotes hepatocyte proliferation and repair, and enhances liver metabolism and detoxification (20). In this experiment, the addition of 0.5% Chinese herbal preparations significantly increased the β-alanine content thereby promoting liver metabolism and improving liver antioxidant function. Amino acids, such as histidine, cysteine, methionine, etc, have antioxidant properties due to the sulphur or amino groups they contain and can be directly or indirectly involved in the antioxidant process. Firstly, it can provide energy and raw materials for the synthesis of other important substances, and secondly, amino acids can be converted into the corresponding amines by decarboxylation, a process that can be involved in the synthesis of neurotransmitters and hormones as well as in the release of energy. For example, glycine and glutamic acid are components of glutathione, the main antioxidant in the liver (21). Glutamine is a precursor to glutathione, which helps cells fight oxidation and scavenge free radicals. It enhances immune responses, reduces inflammation and cellular damage, and modulates antioxidant and anti-inflammatory pathways (9). Glutathione-mediated biotransformation in the liver is a well-known detoxification process for the elimination of small xenobiotics (22). GSH is involved in numerous cellular processes, such as protein folding, safeguarding protein thiols from oxidation and cross-linking, degradation of proteins containing disulfide bonds, regulation of the cell cycle and proliferation, metabolism of ascorbic acid, apoptosis, and iron death (23). In this experiment: oxidized glutathione and reduced glutathione were significantly higher in the liver of the test group than in the control group. It indicates that the addition of herbal preparations can improve the antioxidant function of the liver. Caffeic acid has a significant effect in the ileum, and likewise in the liver. It has been shown that dietary addition of caffeic acid significantly reduced nickel-induced lipid peroxidation and restored antioxidant defense levels in rat liver. These results demonstrate the physiological relevance of caffeic acid and its antioxidant effects in body (24). The addition of 0.5% Chinese herbal preparations in this experiment increased the content of caffeic acid in the liver thereby increasing the antioxidant function of the liver.

The impact of the gut microbiota on human health has been recognized as important and the liver is one of the key organs affected by the gut microbiota. The influence of gut flora on the liver is obvious, but the liver can also influence gut flora in a number of ways. This interaction constitutes the gut-liver axis (25). It has been shown that the higher the level of the *Firmicutes*, the less inflammation there is. Acetylpropionic acid can be broken down by *Firmicutes* into short-chain fatty acids such as acetic acid and propionic acid. These short-chain fatty acids can be absorbed into the bloodstream and then pass through the portal vein to the liver where they are finally used for gluconeogenesis in the liver (26). Studies have shown that dihydromyricetin (DMY), a natural flavonoid, may alleviate NASH by decreasing hepatotoxicity, modulating lipid metabolism, and increasing intestinal probiotics. Flavonoids in herbal medicine were found to increase the abundance of intestinal flora, protect the gastrointestinal tract and the liver, and contribute to the body’s antioxidant capacity.*Christensenellaceae_R_7_ group* is associated with energy metabolism and inflammation, contributing to gut flora balance and nutrient absorption (27). *lachnospiraceae_nk3a20_groups* has compounds with antioxidant and anti-inflammatory properties. Some studies have shown that the higher relative abundance of *lachnospiraceae_nk3a20_groups* may be beneficial to reduce the incidence of diarrhoea in rabbits (28). The addition of Chinese herbal preparations in this experiment could significantly increase the abundance of *lachnospiraceae_nk3a20_groups*, and We can know that Poria cocos in herbal preparations can improve antioxidant in Hu sheep. The *Eubacterium hallii group*, a component of the intestinal microbiota, was observed to produce butyric acid, a short-chain fatty acid. This acid plays a vital role in the regulation of intestinal flora, maintenance of intestinal health, and provision of beneficial effects on intestinal well-being (29). The inclusion of 0.5% of the Chinese herbal preparation in the diet resulted in elevated levels of *Eubacterium hallii group bacteria* in the intestines, consequently exerting a protective influence on the intestinal tract. Several studies have demonstrated the efficacy of Seven-flavor Baijusan, an herbal formulation, in treating diarrhoea of diverse etiologies. This formulation exerts its antidiarrheal properties by modulating the composition of the intestinal microbiota. Notably, the cumulative glycosides present in Seven-flavored Atractylodes Macrocephalae exhibit a targeted influence on select intestinal bacteria and bile acids (30). Saponins are a large class of amphiphilic glycosides, saponins are saponins released by the intestinal flora, which have a variety of biological activities, such as promotion of absorption, antioxidant, anti-inflammatory, and anticancer activity also helps to explain the mechanism of *in vivo* bioactivity of saponins as well as the prevention of several chronic diseases (31). Our findings suggest that the addition of herbal preparations led to an increase in the presence of the *Firmicutes* and *Mycobacterium anisopliae*, which in turn led to an increase in the abundance of γ-commercial and bile acids. Based on these observations, it was demonstrated that polysaccharides and saponins in Chinese herbal preparations can ameliorate diarrhoea in Hu sheep. Furthermore, study demonstrated that ursodeoxycholic acid effectively accelerates the frequency of bile acid recycling within the human body (32). Synthesis of lipocholic acid, deoxycholic acid and their derivatives by intestinal flora serves as a mechanism to regulate signaling molecules in immune cells, including DCs, macrophages, Th1 cells and Th17 cells. The function of these immune cells is closely linked to immune and antioxidant responses (33). In this study, the addition of Chinese herbal preparations led to an increase in the abundance and subsequent content of goose deoxycholic acid, bile acids and deoxycholic acid within the phylum Hautchettia, Mycobacterium anthropophilum, Actinobacteria and Ascomycetes. It can be demonstrated that the use of Chinese herbal preparations enhances the digestion of nutrients, antioxidant processes, promotes the diversity of intestinal flora, and strengthens the immune function of the organisms in hooves.

## Conclusion

In this study, we found that the addition of Chinese herbal preparations resulted in higher content of amino acids and their metabolites, organic acids and their metabolites, bile acids, and increased abundance of enterohepatic flora (*Firmicutes*, and *Bacteroidota*) in the liver and ileum of the Hu sheep. This suggests that the addition of Chinese herbal preparations can promote the digestion, absorption and metabolism of nutrients in the ileum and liver, as well as improve the antioxidant and immune effects on the organism, promote gut-liver circulation, regulate the abundance of gut-liver flora, and increase the resistance to diarrhoea in Hu sheep. The best effect is when the amount added is 0.5 percent.

## Data Availability

The original contributions presented in the study are included in the article/**Supplementary Material**. Further inquiries can be directed to the corresponding authors.
