# Supplementary material for: Supplementation with Chinese herbal preparations protect the gut-liver axis of Hu sheep, promotes gut-liver circulation, regulates intestinal flora and immunity
Source: Front Immunol. 2024 Nov 13;15:1454334. doi: 10.3389/fimmu.2024.1454334 (PMC11599181; doi:10.3389/fimmu.2024.1454334)
Supplement: Supplementary file 1 [file DataSheet1.docx]

**
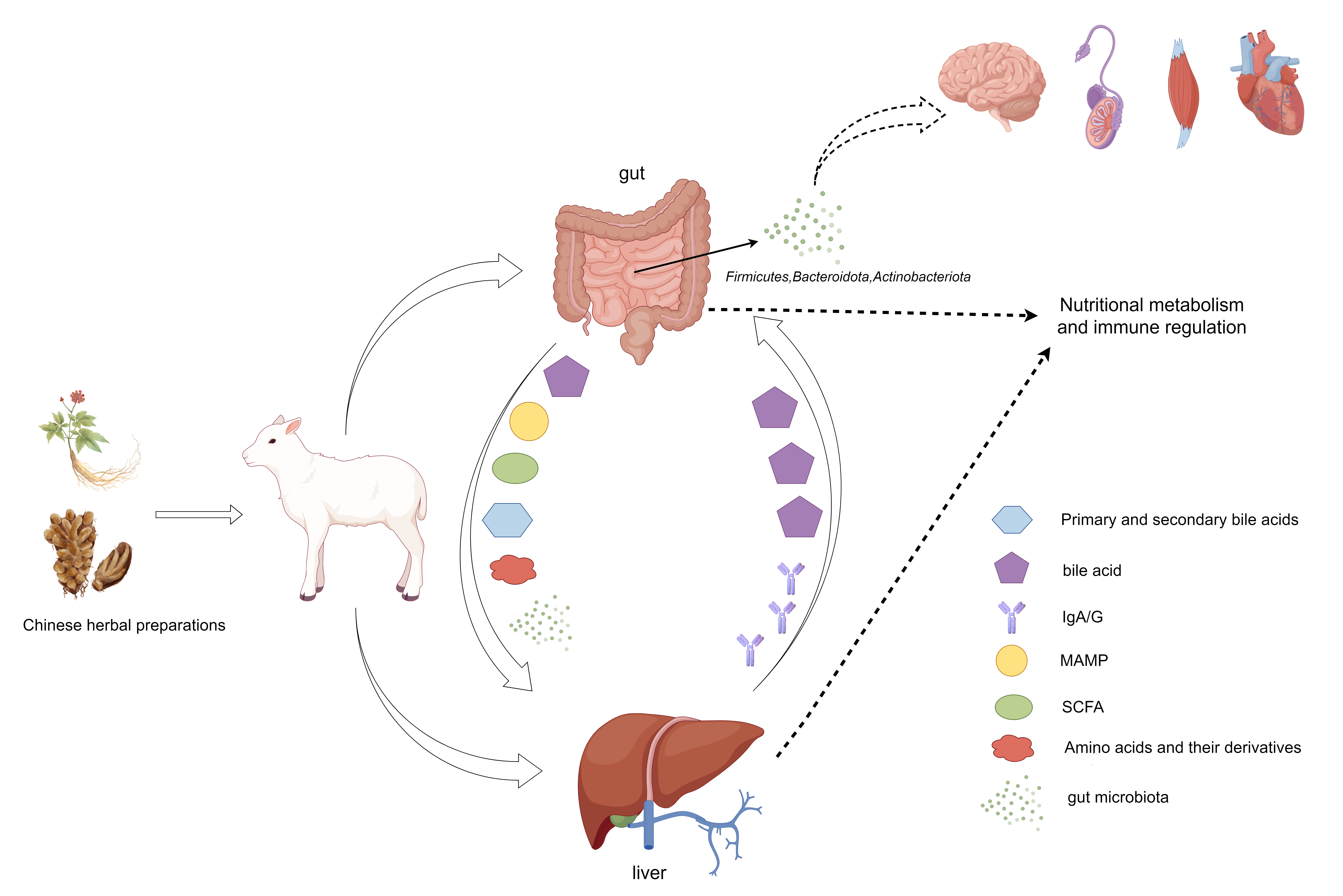
**

**FIGURE** **S1** The mechanism of the gut-liver axis


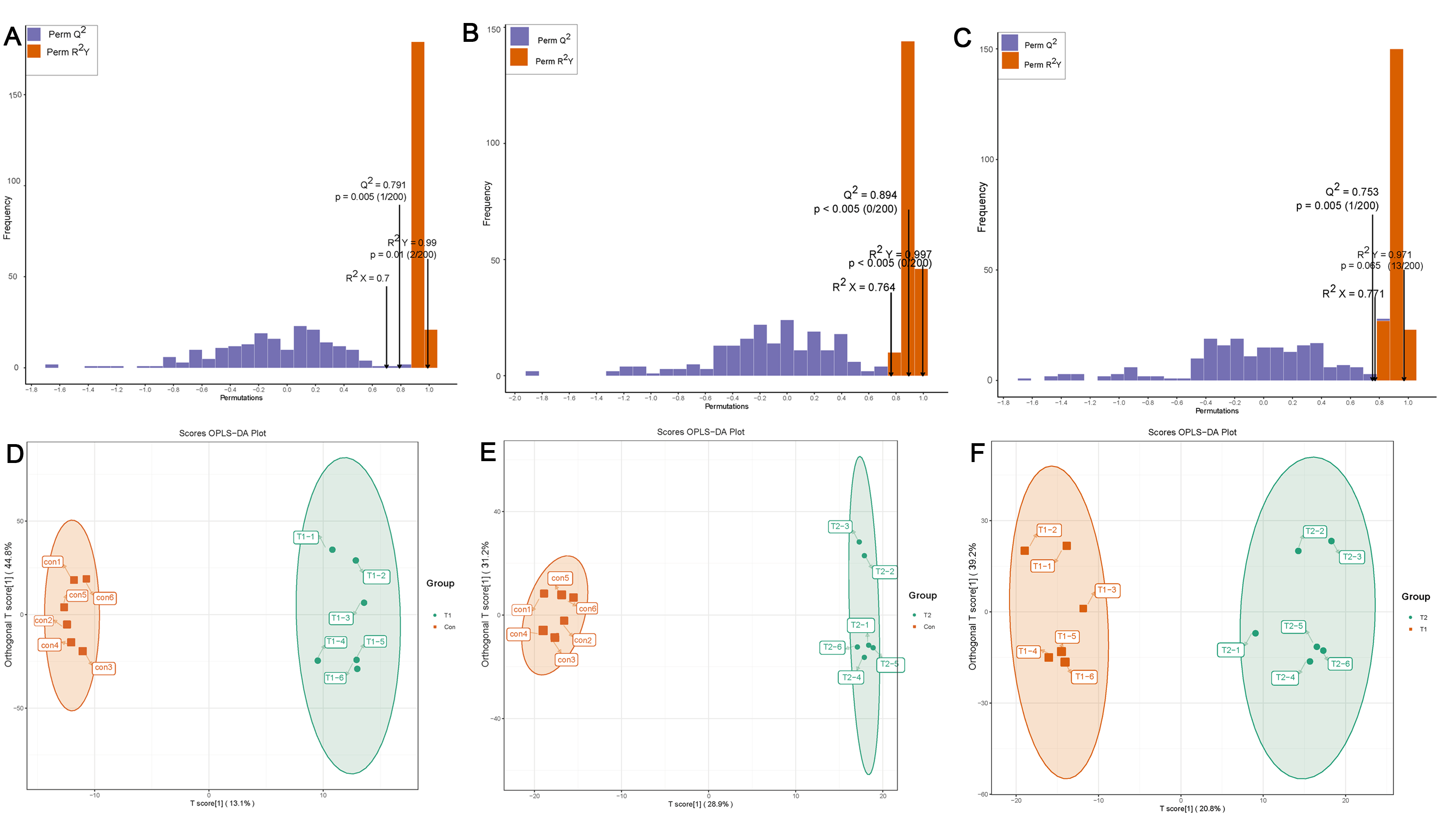


**FIGURE S2** OPLS-DA score plots and model validation plots plotting metabolomics data of different ratios of Chinese herbal preparations on ileum. (A and D) OPLS-DA score plots and model validation plots for Con group vs T1 group, respectively. (B and E) OPLS-DA score plots and model validation plots for Con vs T2 group, respectively. (C and F) OPLS-DA score plots and model validation plots for T1 vs T2, respectively.


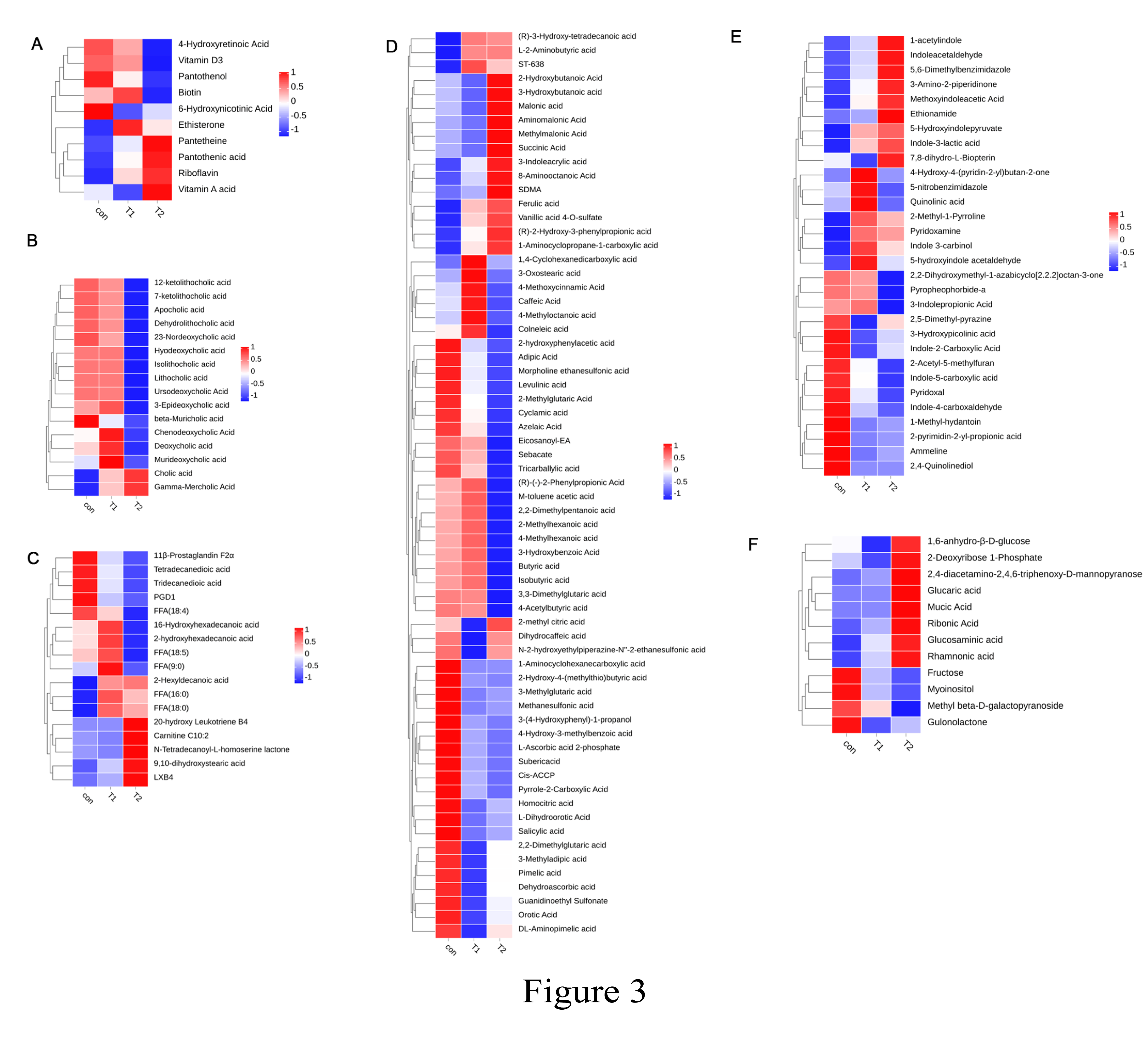


**FIGURE S3.** Distribution of differential metabolite accumulation profiles detected in ileum supplemented with different proportions of Chinese herbal preparations. (A) Coenzymes and vitamins; (B) fatty amides; (C) organic acids and their derivatives; (D) bile acids; (E) heterocyclic compounds; (F) carbohydrates. The normalization heat map shows the mean values of the content of each metabolite in the ileum after the addition of different proportions of Chinese herbal preparations. Six independent replicates were performed for each stage. The colors indicate the proportional content of each identified metabolite as determined by the mean peak response area normalized to the R scale. Red represents up-regulation and blue represents down-regulation.





**FIGURE S4.** OPLS-DA score plots and model validation plots of metabolomics data of different herbal preparations on the liver of Hu sheep. (A and D) are OPLS-DA score plots and model validation diagram for Con and T1, respectively. (B and E) OPLS-DA score plots and model validation diagram for Con and T2 groups. (C and F) OPLS-DA score plots and model validation diagram for T1 and T2.


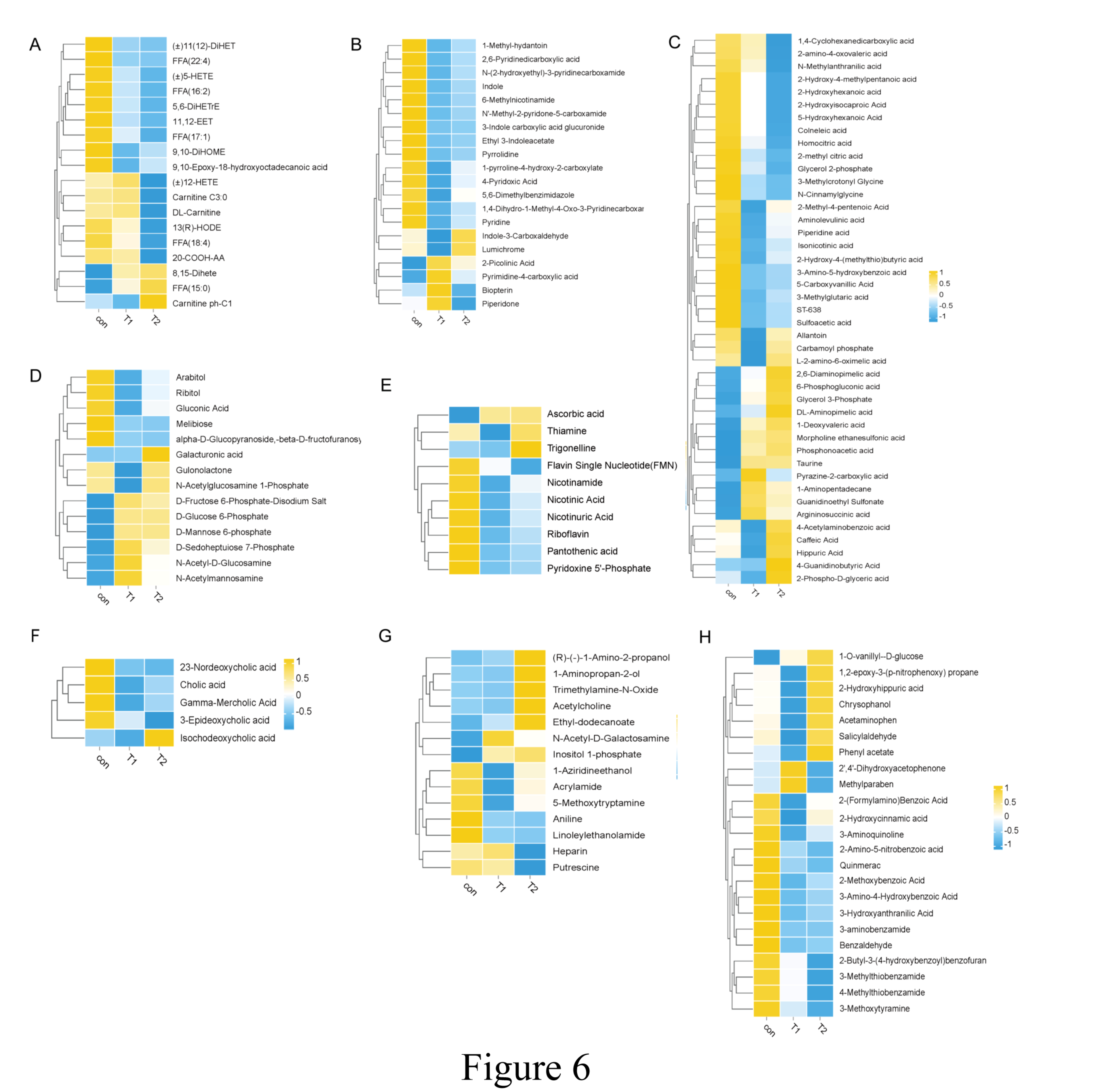


**FIGURE S5.** Cumulative distribution of different metabolites detected in livers supplemented with different proportions of Chinese herbal preparations. (A) Fatty amides; (B) Heterocyclic compounds; (C) Organic acids and their derivatives; (D) Carbohydrates; (E) Coenzymes, vitamins; (F) Bile acids; (G) Alcohols, amine metabolites; (H) Benzene and its derivatives. Normalized Heatmap showing the mean values of various metabolite contents in the liver after addition of different proportions of Chinese herbal preparations. Six independent replicates were performed for each stage. The colors indicate the proportional content of each identified metabolite as determined by the mean peak response area normalized to the R scale. Yellow represents up-regulation and blue represents down-regulation.
