# Supplementary material for: Supplementation with Chinese herbal preparations protect the gut-liver axis of Hu sheep, promotes gut-liver circulation, regulates intestinal flora and immunity
Source: Front Immunol. 2024 Nov 13;15:1454334. doi: 10.3389/fimmu.2024.1454334 (PMC11599181; doi:10.3389/fimmu.2024.1454334)
Supplement: Supplementary file 2 [file Table1.docx]

**Table S1** Venn diagram shared differential metabolites

| Compounds | Class1 | Con | T1 | T2 | Log2FC（ConvsT1） | Log2FC（ConvsT2） | | Log2FC（T1vsT2） |
| --- | --- | --- | --- | --- | --- | --- | --- | --- |
| 2-hydroxyphenylacetic acid | Organic acid and Its derivatives | 567091.12 | 271834.45 | 120277.0489 | -1.06085 | -2.23722 | -1.17637 | |
| 3,3',5-Triiodo-L-Thyronine | Hormones and hormone related compounds | 96927.11 | 169505.36 | 36923.33007 | 0.80636 | -1.39237 | -2.19873 | |
| 3-Chloroaniline | Benzene and substituted derivatives | 41986980.97 | 44084487.01 | 45752106.63 | 0.07033 | 0.12390 | 0.05357 | |
| 6-Methylaminopurine | Nucleotide and Its metabolites | 603526.19 | 203922.08 | 107821.6345 | -1.56540 | -2.48477 | -0.91937 | |
| Dehydroascorbic acid | Organic acid and Its derivatives | 486241.27 | 180231.66 | 335430.6368 | -1.43182 | -0.53566 | 0.89616 | |
| L-Thyroxine | Hormones and hormone related compounds | 100121.95 | 141076.36 | 41836.75312 | 0.49472 | -1.25892 | -1.75363 | |
| Lys-Ala | Amino acid and Its metabolites | 161383.13 | 23067.90 | 58006.98355 | -2.80653 | -1.47619 | 1.33034 | |
| beta-Muricholic acid | Bile acids | 13320007.11 | 5929907.91 | 1035581.099 | -1.16751 | -3.68508 | -2.51757 | |

Mean values of peak areas for each metabolite are listed. Shared differential metabolites were identified using thresholds of VIP ≥ 1 and FC ≥ 2 or ≤ 0.5 for ileus with different proportions of herbal preparations.

**Table S2** Venn diagram shared differential metabolites

| Compounds | Class I | Con | T1 | T2 | Log2FC（T1vsCon） | Log2FC（T2vsCon） | Log2FC（T2vsT1） |
| --- | --- | --- | --- | --- | --- | --- | --- |
| 1-Methyl-hydantoin | Heterocyclic compounds | 718580.09 | 205832.88 | 319981.58 | -1.803675479 | -1.167160098 | 0.636515381 |
| 2-Phospho-D-glyceric acid | Organic acid and Its derivatives | 264029.52 | 187358.03 | 404690.77 | -0.494901467 | 0.616120685 | 1.111022152 |
| Asp-Lys | Amino acid and Its metabolites | 241748.93 | 161089.96 | 192009.58 | -0.5856429 | -0.332331203 | 0.253311697 |
| Isochodeoxycholic acid | Bile acids | 16767.35 | 4891.06 | 58246.05 | -1.777435454 | 1.796505419 | 3.573940873 |
| Lys-Asp | Amino acid and Its metabolites | 241748.93 | 161089.96 | 192009.58 | -0.5856429 | -0.332331203 | 0.253311697 |
| S-(Methyl)glutathione | Amino acid and Its metabolites | 2753094.58 | 4663726.17 | 3612437.64 | 0.760428907 | 0.391918508 | -0.368510399 |

Mean values of peak areas for each metabolite are listed. Shared differential metabolites were identified using thresholds of VIP ≥ 1 and FC ≥ 2 or ≤ 0.5 for ileus with different proportions of Chinese herbal preparations.
